# T follicular regulatory cells: Guardians of the germinal centre?

**DOI:** 10.1111/sji.12942

**Published:** 2020-09-30

**Authors:** Cecilia Fahlquist Hagert, Søren E. Degn

**Affiliations:** ^1^ Department of Biomedicine Aarhus University Aarhus C Denmark

**Keywords:** autoimmunity, germinal centres, T follicular regulatory cells

## Abstract

It is a central tenet of the clonal selection theory, that lymphocyte repertoires are tolerized to self‐antigens during their ontogeny. Germinal centres are the sites in secondary lymphoid tissues where B cells undergo affinity maturation and class‐switching to produce high‐affinity antibodies. This process is crucial, both in our ability to mount protective humoral responses to infections and to vaccinations, but it is also involved in untoward reactions to self‐antigens, which underlie autoimmunity. The process of affinity maturation poses a significant challenge to tolerance, as the random nature of somatic hypermutation can introduce novel reactivities. Therefore, it has been a long‐standing idea that mechanisms must exist which limit the emergence of autoreactivity at the germinal centre level. One of these mechanisms is the requirement for linked recognition, which imposes on B cells a dependence on centrally tolerant T follicular helper cells. However, as linked recognition can be bypassed by adduct formation of autoantigenic complexes, it has been an appealing notion that there should be an additional layer of dominant mechanisms regulating emergence of autoreactive specificities. About a decade ago, this notion was addressed by the discovery of a novel subset of T regulatory cells localizing to the germinal centre and regulating germinal centre B‐cell responses. Here, we detail the progress that has been made towards characterizing this T follicular regulatory cell subset and understanding the functions of these ‘guardians of the germinal centre’.

## 1 INTRODUCTION

Germinal centres (GCs) are the sites in secondary lymphoid tissues where B cells undergo affinity maturation to produce high‐affinity antibodies. A central aspect of the GC reaction is its regulation to protect against tissue damage and autoimmunity. This has long been thought to rely on the requirement for linked recognition, whereby T follicular helper cells (Tfhs) license B‐cell reactivities in T‐dependent responses. However, this mechanism is not fail‐safe. Indeed, many autoimmune diseases, such as systemic lupus erythematosus (SLE) and rheumatoid arthritis, are characterized by the presence of affinity‐matured, class‐switched antibodies to autoantigens.^1^ Nearly a decade ago, it was discovered that a unique subset of T regulatory cells, the T follicular regulatory cell (Tfr), localizes to GCs and exerts a dominant suppressive level of control on the GC reaction.^2^, ^3^, ^4^ Hence, control of GC responses is, as with most immune mechanisms, multi‐layered. It appears that the balance between Tfh and Tfr regulates normal foreign antigen–directed GC responses and that failure of Tfr may contribute to humoral autoimmunity.^5^ Here, we discuss the current state of knowledge regarding GC function and its regulation by Tfrs.

## 2 GERMINAL CENTRES

Germinal centres are formed upon antigenic stimuli in all peripheral lymphatic organs, such as lymph nodes (LN), spleen and Peyer's patches. GCs induced in response to a transient pulse of antigen typically arise around day 5‐6, peak at about 12‐14 days and wane ~3 weeks.^6^ Some lymphatic organs are in constant contact with foreign antigens and have a constant expression of GCs, for example the Peyer's patches of the small intestine and the mesenteric LN lining the gut, both important in controlling the commensal flora. It is unclear whether GCs in these tissues are continuously being replaced or if individual GCs are continually present. In support of the latter possibility, it has been shown that pre‐existing GCs can be co‐opted by novel naïve clones as well as memory B cells, with new and old specificities,^7^, ^8^, ^9^ although recent observations have cast doubt upon the efficacy of memory B‐cell reentry into GC.^10^

**Figure 1:**
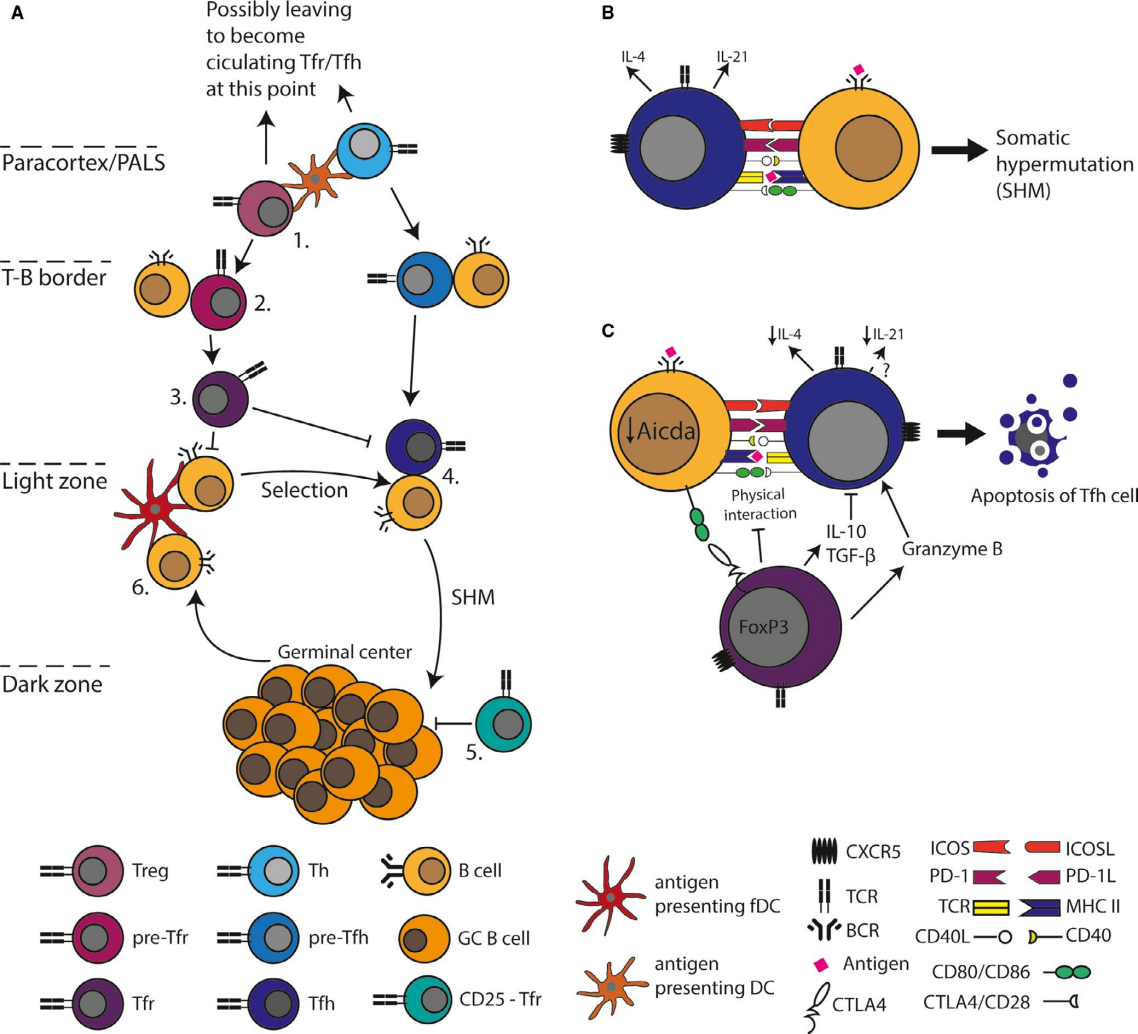
A, Prior to entering the germinal centre (GC), both Tregs and T helper cells (Th) need to interact with a dendritic cell (DC) initiating a pre‐Tfr and a pre‐Tfh programme, respectively (1). This allows them to enter the follicle where interaction with B cells further drives commitment to Tfr and Tfh fates, respectively (2). The role of Tfr in the GC is to regulate the B and Tfh cells (3), whereas Tfhs decide which B cells should undergo another cycle of somatic hypermutation (SHM) (4). There is some evidence that CD25‐ Tfrs license GC B‐cell exit from the dark zone. GC B cells interact with antigen‐presenting follicular dendritic cells in the light zone. If they display specificity for the correct antigen, they get selected by Tfh to undergo SHM again until they attain high affinity, at which point they leave the germinal centre as plasma cells. B, The cross‐talk needed between a B cell and a Tfh cell to promote SHM. C, The signal pathways in which Tfrs are thought to regulate B and Tfh cells

The first step in GC formation is the activation of B cells by soluble antigen or antigen retained by follicular dendritic cells in the follicle, and the concomitant activation of naïve CD4 T cells by antigen‐presenting dendritic cells, initiating their differentiation into Tfh (Figure 1A). The activated B cells downregulate the chemokine receptor CXCR5, responsible for their localization in the follicle, and upregulate the chemokine receptor CCR7 increasing their responsiveness to chemotactic signals provided by CCL‐19 and CCL‐21 produced by stromal cells in the paracortical T‐cell zone. Conversely, the nascent Tfhs downregulate CCR7 and upregulate CXCR5, directing them to migrate towards the follicular CXCL13 gradient to the T‐B border of the follicle. Here, initial direct interaction with B cells occurs; if productive, this results in formation of an extrafollicular primary focus yielding plasma blasts and short‐lived plasma cells, and the initiation of a GC.^11^ The productive interaction strengthens the Tfh commitment, and B cells and Tfh co‐migrate into the follicle, where B cells begin to proliferate vigorously, forming the GC.

While it was initially thought that B cells undergo isotype class‐switching in GCs, recent data have demonstrated that this most likely occurs in primary foci, prior to GC entry and seeding.^12,13^ It has been found that on the order of hundreds of clones can seed an incipient GC.^14^ In GCs, B cells cycle between the dark zone (DZ) and the light zone (LZ). The DZ is formed by rapidly proliferating B cells, termed centrocytes, observable by light microscopy as dense, dark areas of high cell density. In the DZ, B‐cell clones expand and undergo somatic hypermutation leading to introduction of mutations in their immunoglobulin V(D)J loci. In the LZ, resulting daughter B cells, each having accrued subtle changes in their B‐cell receptor, scan follicular dendritic cells for antigen. Most mutations will reduce the affinity for antigen, abrogate antigen binding altogether or introduce changes in framework regions which preclude correct folding or pairing of immunoglobulin chains, and cells carrying such changes are lost by apoptosis. B cells that retain or increase antigen affinity can take up antigen through their B‐cell receptor, present antigen‐derived peptides to cognate Tfh, and are stimulated to return to the DZ for another round of division and hypermutation^11^, ^15^ (Figure 1A). In this iterative Darwinian process of diversity generation and selection based on affinity, B‐cell clones which recognize antigen with increasingly high affinity are selected and expanded. Already early on in the initial response and early GC, some responding B cells give rise to long‐lived memory B cells that can subsequently be found in multiple tissues and that have been shown to be able to transfer memory responses to naïve recipients. Eventually, high‐affinity clones give rise to long‐lived plasma cells, which home to the bone marrow, where they persist in an antigen‐independent manner and constitutively secrete high‐affinity antibodies conferring serological memory.^16^

## 3 TFH CELLS

Tfhs have been extensively studied in the context of foreign antigen responses and are thought to control GC B‐cell cycling between LZ and DZ in a peptide‐major histocompatibility complex II (MHCII)–directed manner^15^ (Figure 1A and B). Tfhs are regulated by the transcription factor Bcl‐6 and characterized as CD4+ CXCR5hi ICOS+ PD‐1+ T cells ^17^ (Table 1). Induction of the Tfh programme in naïve CD4^+^ T cells is initiated through initial contact with antigen‐presenting dendritic cells. The pre‐Tfhs migrate to the T‐B border and contact cognate B cells, eliciting further signals that fix the Tfh transcriptional programme. The Tfhs subsequently localize to the GC and make transient contacts with B cells^18^ and if stimulated by cognate peptide bound to MHCII they flux calcium and upregulate interleukin (IL)‐4, IL‐21 and CD40L to induce B‐cell return to the DZ. Tfhs distribute among all GCs in LN and can move between the GC and interfollicular regions, but do not enter the circulation.^18^

**Table 1:** Similarities and differences in markers of T follicular regulatory cells (Tfrs), T follicular helper cells (Tfhs) and conventional T regulatory cells (Tregs). CD25‐ GC‐Tfr cells have been reported to control GC

	Pre‐Tfr	GC‐Tfr	Pre‐Tfh	GC‐Tfh	Treg	References
FoxP3	+	+	−	−	+	[3, 25, 55]
Blimp‐1	High	Low	−	−	+	[3, 25, 55, 56]
CXCR5	+	High	+	High	−	[3, 25, 33, 42, 55]
PD‐1	+	High	+	High	+	[3, 25, 33, 55]
ICOS	High	High	+	High	In some	[3, 17, 25, 33]
Bcl‐6	+	High	+	High	−	[25, 33]
CD25	+	−	−	−	+	[25, 55, 57]
CTLA‐4	Low	High	+	+	+	[25, 30, 36]
IL10	+	Low		+	High	[25, 33, 43]

The notion that Tfhs directly control GC B‐cell cycling between the DZ and LZ was recently challenged by observations in a foreign immunogen setting suggesting that the fitness of GC B cells does not titrate with peptide‐MHCII density.^19^ In this study, Yeh *et al* found that peptide‐MHCII density impacted the ability of B cells to *enter* the GC reaction, but once in the GC, B cells with a 50% reduction in peptide‐MHCII density competed efficiently with wild‐type B cells. This notion fits well with prior observations by us, that once tolerance is broken and autoreactive GCs are initiated, they evolve clonally in a manner comparable to foreign antigen–elicited GCs.^6^, ^20^ Potentially this suggests that protoautoreactive B cells are incompletely controlled by a strict requirement for linked recognition once they have entered GCs, although an alternative explanation could also be breakage of tolerance at the level of T cells.

Taken together, these observations imply the necessity of auxiliary tolerogenic mechanisms controlling physiological GC responses and preventing break of tolerance at the GC stage. Indeed, it has become clear that an additional T follicular cell subset is centrally involved in regulating GCs, namely the Tfr.

## 4 TFR ONTOGENY AND PHENOTYPE

Although it had been previously noted that Tregs have the capacity to enter follicles and suppress GC responses,^21^, ^22^, ^23^ Tfrs were only recognized as a distinct cell subset in 2011.^2^, ^3^, ^4^ Tfrs are characterized by expression of CXCR5, PD‐1, ICOS, CTLA‐4 and the transcription factors FoxP3, Blimp‐1, and Bcl‐6^2^, ^3^, ^4^ (Table 1). Bcl‐6 is essential for formation of Tfr, while Blimp‐1 expression regulates the population size.^3^, ^24^ Expression of CD25, the alpha‐chain of the high‐affinity IL‐2 receptor, is common on Tregs outside the T‐B cell border and on Tfr outside the GC, but has been reported to be downregulated on Tfr within the GC (GC‐Tfr)^22^, ^25^, ^26^, ^27^ (Table 1). This suggests that, similar to Tfh, Tfrs undergo at multistep differentiation process, culminating in a GC‐Tfr phenotype accompanied by downregulation of CD25. This shields them from IL‐2 signalling, which would otherwise drive upregulation of Blimp‐1 and inhibit high‐level expression of Bcl‐6. Although some of their Treg characteristics are downregulated, they retain key features such as expression of FoxP3 and CTLA‐4.^22^, ^25^, ^27^ However, more studies are required to fully determine the phenotype and functional properties of the CD25‐ cells, as a recent report has demonstrated that Tfrs which lose FoxP3 expression and suppressive capacity (so‐called ex‐Tfr) are also characterized by CD25 downregulation.^28^

Induction of Tfr requires co‐stimulation through CD28 and ICOS^3^, ^29^ and is restricted by co‐inhibitory signals through PD‐1 and CTLA‐4.^29^, ^30^, ^31^ Initiation of the Tfr programme is orchestrated by two helix‐loop‐helix family members, inhibitor of differentiation (Id) 2 and Id3. T‐cell antigen receptor (TCR)–driven signalling downregulates Id3, eliciting a Tfr‐specific transcription signature. However, sustained decreases in Id2 and Id3 interfere with proper development of Tfr.^32^ It is believed that TCR signal strength is a key element in Tfr generation, but this aspect remains incompletely understood.^33^ Although Tfr can be found that are specific for an immunizing foreign antigen,^34^ the TCR repertoires of Tfh and Tfr have been demonstrated to be distinct from one another in a foreign antigen setting.^35^

As can be seen in Table 1, Tfrs share characteristics with both Tregs and Tfh. Importantly, Tfrs express the same homing markers as do Tfh, localizing both subsets to the peripheral lymphoid organs and, upon activation, to the GC. Indeed, Tfrs are often distinguished from Treg by expression of CXCR5. It was found that NFAT2 is essential for upregulation of CXCR5 in Tfr, and ablation of NFAT2 reduced levels of Tfr in the follicular T‐cell population and caused exacerbation of the GC reaction.^5^ However, recent data suggest that CXCR5 is not necessary for localization of Tfr to GCs, indicating that CXCR4 or other redundant mechanisms might compensate for loss of CXCR5.^36^

## 5 TFR CELL HOMEOSTASIS

Tfr development is regulated by the Tfh‐produced cytokine IL‐21, which inhibits the proliferation of Tfr through Bcl‐6‐mediated suppression of IL‐2 responsiveness via downregulation of CD25.^37^ Conversely, Tfrs control the expansion and function of Tfh. Taken together, this creates an important cross‐regulation between Tfr and Tfh. The dynamic balance between the two subsets is thought crucial to permissive GC responses during acute inflammation, when Tfhs dominate, and central to restricting the GC reaction once the response has peaked and Tfrs resume control. Interestingly, the IL‐2 peak during influenza virus infection promotes the expression of Blimp‐1 in conventional Treg, thus suppressing Bcl‐6 expression and precluding the development of Tfr. However, Blimp‐1 is not the sole regulator of Bcl‐6, since Bcl‐6 was also repressed in Blimp‐1‐deficient CD25hi Treg. Once infection resolved, wild‐type CD25hi Treg cells downregulated CD25 and upregulated Bcl‐6, thus differentiating into Tfr. Thus, this study indicated an important role for Tfr to re‐establish and sustain B‐cell tolerance subsequent to a transient permissive environment during influenza infection.^26^ That view was, however, challenged by a recent study, which used a novel inducible Tfr deleter mouse that enabled precise analyses of kinetics of GC regulation, and found that Tfr cells mainly regulate the incipient GC response, whereas they have little to no effect once the GC is formed.^38^ This raises the question of how such a purported restriction of Tfr activities to the pre‐ to early GC stage could integrate with the previously discussed possibility for reutilization of pre‐existing GCs by naïve and memory clones. Notwithstanding such mechanistic considerations, taken together, the growing knowledge of the basic inner workings of the Tfr subset firmly establishes their central functions in homeostatic regulation of GC responses.

## 6 CIRCULATING TFR MEMORY‐LIKE CELLS

A central aspect of immune function is the phenomenon of ‘memory’, based on the persistence of specialized memory lymphocytes after resolution of an immune response, which can be reactivated upon repeated exposure to the same challenge.^39^ In addition to being present in lymphoid organs, memory‐like Tfrs are also present in the circulation. Like in the LN, Tfrs in the blood inhibit antibody production. Indeed, effector LN Tfr from the circulation suppressed both the activation of Tfh and production of Tfh cytokines, including IL‐21. Class‐switch recombination and B‐cell activation were also regulated by Tfr, independent of specific antigens. However, the capacity of circulating Tfr seems lower compared to those residing in lymphoid organs. It has been shown that these Tfrs have an immunological memory‐like function. The circulating Tfrs develop with similar kinetics regardless of whether they are destined for LNs or blood.^29^, ^40^ A hypothesis is that Tfrs have two fates after antigen presentation by dendritic cells. One subset, fated to become circulating memory‐like Tfr, expressing medium‐to‐low CXCR5 and low CD69 will exit the LN via S1P gradients while Tfr with high CXCR5 and CD69 expression will follow CXCL13 gradients to the B‐cell zone and regulate the GC response. Indeed, the pre‐Tfrs that enter the lymphoid organs exhibit a phenotype similar to that of circulating Tfr. This indicates that the memory‐like Tfr phenotype is acquired during differentiation, prior to further differentiation towards GC‐Tfr cells.^40^

## 7 TFR CELLS IN CONTROL OF GCS

Deletion of Tfr leads to loss of control of GCs, which expand due to an increase in total numbers of both GC B cells and Tfh.^2^, ^3^ Furthermore, lack of Tfr leads to increased antigen‐specific IgM and IgG antibodies in immunized mice. Several studies have shown a lowering of the affinity of antibodies produced in a Tfr‐deficient environment. For example, Kawamoto et al^41^ found that Tfrs are essential for the formation of high‐affinity IgA antibodies in Peyer's Patch GCs, while Linterman et al^3^ found an increased outgrowth of non‐specific clones in cutaneous LN in a foreign immunogen setting. The likely explanation for these findings is that Tfrs are crucial to focus the GC response by restricting Tfh and B‐cell specificities, giving rise to higher affinity antibodies. Conversely, Chung et al^2^ found an increase in antibody affinity in the absence of Tfr. Taken together, these findings suggest that Tfrs have the ability to restrict antibody responses, but in a physiological setting, this likely serves to specifically prevent the outgrowth of divergent and potentially autoreactive clones. However, a recent report found that Tfr can actually support antiviral GC responses through localized production of IL‐10, which acted on GC B cells to promote DZ cycling.^42^ Along a similar line, Xie et al found that Tfrs regulate the outgrowth of an abnormal subset of granzyme B expressing Tfh, which otherwise exert a cytotoxic effect on GC B cells and hence limit GC responses.^43^ Further studies are needed to clarify the precise nature of these potential activities. Although a more nuanced picture of the roles and functions of Tfr in regulating GC reactions may be emerging, here we focus on the more well‐established suppressive functions of Tfr.

## 8 MECHANISM OF TFR CONTROL OF GCS

Tfr may restrict GC reactions either indirectly, through effects on Tfh or directly through effects on B cells (Figure 1C). It has been shown that Tfrs potently suppress Tfh activation, both in terms of their proliferation and the production of key Tfh cytokines such as IL‐4 and ‐21.^40^ However, it remains unclear exactly how they exert these suppressive effects, which could be mediated by alterations in the cytokine milieu or direct contact‐dependent actions. Tfr may produce the immunosuppressive cytokine TGF‐β, which has been shown to suppress Tfh function and prevent B‐cell autoreactivity.^44^ It is believed that peripheral Tregs may restrain T‐cell expansion by soaking up excessive IL‐2, hence restricting availability of this important growth and survival signal.^45^ However, as Tfh differentiation is directly inhibited by IL‐2,^46^ this mechanism seems an unlikely constituent of Tfr functions in the GC. Conversely, during influenza infection, it has been found that Treg can indirectly promote Tfh‐cell differentiation and in turn the GC response, by limiting T‐cell exposure to IL‐2.^26^, ^47^ Again, it seems unlikely that Tfr would mirror this effect, at least within the GC, as they downregulate CD25 upon GC entry.^25^ Tfrs have been shown to be able to control the magnitude of the GC response through CTLA‐4.^30^, ^31^ However, whereas Tregs were found to directly inhibit B‐cell expression of CD80 and CD86, essential for Tfh formation, this mechanism did not appear to be the basis of the suppressive role of CTLA4 expressed on Tfr. Based on an earlier report that CD4^+^ CD25^+^ Tregs are capable of directly killing B lymphocytes in vitro in a perforin‐dependent manner, it has also been suggested that Tfr may directly kill B cells, although it was noted that Tfrs express lower levels of granzyme B than conventional Treg.^3^ Based on co‐culture assays, Tfr cells have furthermore been found able to suppress the metabolic capacity of B and Tfh cells.^48^ A number of studies have also begun characterizing the impact of dysregulation of Tfr in autoimmune conditions.

## 9 TFR CELLS IN AUTOIMMUNITY

Relatively little data have emerged regarding the specific behaviour of Tfr in autoimmune GCs, but numerous studies have linked Tfr deficiency with autoimmunity. A priori, one would expect a major role for Tfr‐mediated GC control specifically in autoimmune disorders characterized by high levels of circulating autoantibodies. Indeed, in SLE, the frequency of circulating Tfr has been shown to be reduced, while the Tfh/Tfr ratio was increased. Interestingly, there was a negative correlation of the frequency of Tfr to disease activity and the titre of anti‐dsDNA antibody. The Tfh/Tfr ratio was positively correlated with disease activity, indicating that the Tfr suppressive effect upon Tfh is lowered in SLE. In successfully treated patients, the frequency of Tfr was increased and the Tfh/Tfr ratio decreased.^49^ In rheumatoid arthritis, the levels of Tfr are increased in patients with stable disease compared to controls in active disease, and even more increased in patients with stable remission.^50^ In agreement with these findings in patients, mice with a complete block in Tfr development, due to Foxp3‐Cre‐driven conditional knock‐out of Bcl‐6, were more prone to develop spontaneous autoimmune disease resembling Sjögren's syndrome, and displayed an exacerbated phenotype in an experimental Sjögren's syndrome model.^51^ However, these mice also exhibited enhanced protection to influenza virus infection, indicating that Tfr did not specifically control autoimmune responses, but exerted a broader control of GC responses, which also limited reactions to foreign antigens.

The role of Tfr in controlling autoimmunity also extends to autoimmune disorders traditionally considered T cell–driven, but where B cells have recently emerged as important contributors to initiation of disease by virtue of their antigen‐presenting capabilities. Hence, Tfrs were recently associated with the development of type 1 diabetes mellitus.^52^ The frequency of Tfr was negatively correlated with the production of clinical autoantibodies in mice and men, and adoptive transfer of Tfr to the NOD‐SCID model prevented diabetes development. It was also shown that multiple sclerosis patients have a decreased frequency and impaired functionality in their circulating Tfr—memory‐like Tfr, which mirror the Tfr found in follicles of the lymphoid organs.^53^

The roles of Tfr cells in autoimmune disease still remain to be fully elucidated, but a more comprehensive overview of the current state of knowledge has been provided elsewhere.^54^ Whether targeting Tfr cells clinically to treat autoimmune disease is a viable option remains to be seen; however, this will most likely depend on individual assessment for each autoimmune disorder.

## 10 OUTLOOK

Many outstanding questions remain regarding the function of Tfr in regulation of GC homeostasis in health and disease. A deeper understanding of the exact molecular mechanisms by which Tfrs regulate the humoral immune response, both indirectly through Tfh and directly through effects on B cells, may begin to unravel this further. Considering the rapid progress that has been made since their discovery only a decade ago, there can be no doubt that the coming years of research into Tfrs will be highly fruitful.

## CONFLICT OF INTEREST

The authors declare no conflict of interest.

## AUTHOR CONTRIBUTIONS

Both authors contributed to drafting and finalizing the manuscript.
